# Mr. Cowley, on Dislocation of the Jaw

**Published:** 1800-12

**Authors:** J. Cowley

**Affiliations:** St. Thomas's Hospital


### *To the Editors of the Medical and Phyfical Journal.*

GENTLEMEN,

THE refult of the following cafe I hope will not be deemed unworthy your attention, and a place in your valuable Journal; defirous of fhowing the junior practitioner the propriety of his attempting to afford relief even in thofe cafes where the endeavours of the more experienced in the profeſſion have been defeated. I am,

GENTLEMEN,

Your's, very reſpectfully,

*St. Thomas's Hoſpital,* J. COWLEY.

*Oct.* 16, 1800.

---

James Flocker, aged 29, of low ſtature, but of robuſt make, was admitted into St. Thomas's Hoſpital on Sept. 25, 1800, with a luxation of the jaw. Said, on the 27th of Auguſt laſt, he retired to bed at his uſual hour, in health; but at three o'clock the next morning, he awoke with a violent pain in the face, attended with much ſtiffneſs, and the mouth in an open, fixed ſtate. The ſame day he applied to two ſurgeons for advice, who both agreeing that it was a luxation of the jaw, attempted its reduction repeatedly, without ſucceſs: the patient remained under their care three weeks; but receiving no relief, he left the country for town, and on the above day applied at St. Thomas's Hoſpital, with an evident luxation of the jaw; yet, in other reſpects, apparently in health. His appetite good; pulſe full and regular; the muſcles of the face in a ſtate of rigidity; and his mouth ſore from the treatment he had received; ſo that the introduction of the finger was attended with much pain. Under theſe circumſtances, I conſidered much depended on my firſt attempt; and Mr. Birch (the ſurgeon under whoſe care he was admitted) having adviſed me to effect as complete a relaxation of the parts as poſſible, before any means were uſed for its replacement, I applied five leeches on each ſide of the face, in the direction of the maſſeter and temporal muſcles, which having dropped off, the after hæmorrhage was much increaſed by placing the patient in the warm bath at 98°, which was gradually increaſed to 106 degrees of heat of Farenheit's thermometer. After the patient had been in twenty minutes, his pulſe ſunk, became irregular, and ſhewed ſymptoms of ſyncope; at this juncture (the patient remaining in the bath) I attempted to preſs on the jaw with my thumbs covered, but finding it impoſſible to act with advantage in this manner, I removed the incumbrance, and introduced them unarmed and preſſing on the jaw as far back as poſſible, beginning with a ſlight preſſure, ſlowly adding the force requiſite, I continued the extenſion downward, with a ſlight inclination forward, for ten minutes; at this time I tried to force back the jaw, and evidently perceived it to be altered in its ſituation; but on its producing ſome pain, the patient forced himſelf from my hands, and the foſſæ receiving the condyles of the jaw, being in a great meaſure filled up with an extravaſated ſubſtance, (this I conſider confider to be the fact, from the jaw not flipping into its place fuddenly, as is generally expected in thefe accidents) it refumed its wrong fituation with the greateft facility: At this time his pulfe began to encreafe, both in fize and fulnefs; I therefore immediately renewed my extenfion as before, and having continued it for ten minutes, again preffed backward, and had the fatisfaction to fee it properly reduced. The parts were now fecured by a bandage, and the patient retired to bed. 26th. He complains of pain in the direction of the temporal mufcles, his pulfe weak, foft, and flow; the fymptoms of irritation are not however fo great as expected, fo that a confinement to bed is unneceffary; he took an aperient draught this afternoon. 27th. His pulfe is increafed in ftrength and fulnefs; the draught procured him three motions, which gave great relief; from this time he may be confidered as in perfect health, capable of mafticating folid food, and in every other refpect as well as before the accident. October 9, he was prefented out of the Hofpital, cured.

